# Prevalence of premenstrual syndrome and premenstrual dysphoric disorder in India: A systematic review and meta-analysis

**DOI:** 10.34172/hpp.2021.20

**Published:** 2021-05-19

**Authors:** Abhijit Dutta, Avinash Sharma

**Affiliations:** ^1^Department of Medical Research and Data Management, Sanjiban Hospital, Howrah, India; ^2^Department of Psychiatry, Central Institute of Psychiatry, Ranchi, India

**Keywords:** Premenstrual syndrome, Premenstrual dysphoric disorder, Prevalence, Systematic review, Meta-Analysis

## Abstract

**Background:** The burden and impact of premenstrual syndrome (PMS) and premenstrual dysphoric disorder (PMDD) is not well characterised among Indian population. Therefore, we conducted this systematic review and meta-analysis to estimate the prevalence of PMS and PMDD among females of reproductive age group living in India.

**Methods:** We searched PubMed, Cochrane Library, Scopus and IndMed for studies reporting the prevalence of PMS and/ or PMDD from any part of India, published from 2000 up to Aug 2020. We performed random-effects meta-analyses evaluated using I^2^ statistic, subgroup analyses, sensitivity analyses and assessed study quality. Estimated prevalence along with 95% confidence intervals (CIs) were reported for each outcome of interest. The quality of each study was evaluated using modified Newcastle Ottawa Scale (NOS). This review was conducted following the standard of Preferred Reporting Items for Systematic Reviews and Meta-Analyses (PRISMA) and Meta-Analysis of Observational Studies in Epidemiology (MOOSE) guidelines. The protocol was registered prospectively in PROSPERO (CRD42020199787).

**Results:** Our search identified 524 citations in total, of which 25 studies (22 reported PMS, and 11 reported PMDD) with 8542 participants were finally included. The pooled prevalence of PMS and PMDD were 43% (95% CI: 0.35-0.50) and 8% (95% CI: 0.60-0.10) respectively. The estimated prevalence of PMS in adolescence was higher and account to be 49.6% (95% CI: 0.40-0.59). The heterogeneity for all the estimates was very high and could be explained through several factors involved within and between studies.

**Conclusion:** This study identified a substantially high prevalence of PMS and PMDD in India. To identify potentially related factors, more focused epidemiological research is warranted. However, noticing the fact of significant prevalence and its potential impact on the population, stakeholders and policymakers need to address this problem at the community and individual level.

## Introduction

Premenstrual disorders occur during the luteal phase of the menstrual cycle and resolve shortly following menstruation. The luteal phase lasts from ovulation to the start of menstruation. Premenstrual disorders are diagnosed depending upon whether the diagnostic criteria followed are from the American College of Obstetricians and Gynaecologists (ACOG)^1^ or from the Diagnostic and Statistical Manual – fifth edition (DSM-5) of the American Psychiatric Association (APA).^2^ Whereas ACOG requires the presence of at least one affective symptom (e.g. angry outbursts, anxiety, confusion, depression, irritability or social withdrawal) and one somatic symptom (e.g. abdominal bloating, breast tenderness or swelling, headache, joint or muscle pain, swelling of extremities or weight gain) for a diagnosis of premenstrual syndrome (PMS)^1^, APA requires only the presence of somatic symptoms.^2^ As for premenstrual dysphoric disorder (PMDD), DSM-5 requires the presence of five symptoms in total, with at least one affective symptom (mood swings, marked irritability, marked depressed mood or marked anxiety) along with other symptoms, which may include somatic symptoms. Certain duration criteria for the symptoms need to be met too.^2^ Symptoms may appear anytime between menarche and menopause. Premenstrual disorders cause significant distress or interfere with work, school or usual social activities and lower quality of life.^3^ These disorders are treatable – selective serotonin reuptake inhibitors (SSRIs) such as sertraline, paroxetine, fluoxetine and escitalopram have been shown to treat both the psychiatric as well as physical symptoms^4^; other medications that have shown benefit include quetiapine (as an adjunct to an SSRI),^5^ oral contraceptives^6^ and calcium supplementation.^7^ Among non-pharmacological treatments, evidence suggests that cognitive behaviour therapy may be helpful.^8^

Although premenstrual disorders are not culture-bound syndromes, cultural factors play an important role in the frequency, intensity and expressivity of symptoms and help-seeking patterns.^2^

The reported prevalence estimates of PMS in India have ranged from 14.3%^9^ to 74.4%.^10^ Similarly, the reported prevalence of PMDD in India has varied widely between 3.7%^11^ to 65.7%.^12^ Factors influencing prevalence estimates include diagnostic criteria or tools used as well as socio-demographic and sub-cultural differences within a diverse country such as India that impact expressivity of symptoms.

Owing to the more or less taboo nature of menstruation in conservative societies as in India, coupled with the traditional gender role subscribed to by females, awareness regarding premenstrual disorders and/or help-seeking behaviour for these disorders has been sub-optimal. Given the treatability of these conditions, proper health policy formulation and implementation to address premenstrual disorders can go a long way in reducing the treatment gap. However, a prerequisite for appropriate policy formulation includes availability of high quality information on which to base effective policy. A meta-analysis of existing studies is a proven method of providing high quality scientific evidence.

To the best of our knowledge, this is the first meta-analysis of prevalence studies of premenstrual disorders in India. The purpose of this study is to provide a better understanding of the epidemiology of PMS and PMDD in India, highlighting inter-regional differences in prevalence, so as to sensitise practising health professionals as well as enabling health planners to allocate scarce health resources commensurate to the scale of the problem.

## Materials and Methods

This systematic review has been conducted to know the estimated prevalence of PMS and PMDD among females of reproductive age group in India. The whole review process has been conducted and narrated in accordance with the Preferred Reporting Items for Systematic Reviews and Meta-Analyses (PRISMA),^13^ and Meta-Analysis of Observational Studies in Epidemiology (MOOSE)^14^ guidelines. The protocol was registered prospectively in International prospective register of systematic reviews (PROSPERO), the Centre for Reviews and Dissemination, University of York (CRD42020199787),^15^ prior to the commencement of the study.

### Search strategy

Four electronic databases namely, PubMed (US National Library of Medicine, National Institutes of Health), Cochrane library, Scopus and IndMed were searched for potential studies from inception up to August 2020. The search was not restricted to the language or publication date. Final search strategy was decided by consensus among the authors.

The following search strategy was used to identify studies in PubMed: (“Premenstrual Syndrome”[Title/Abstract] OR premenstrual syndrome[MeSH Terms] OR premenstrual syndromes[MeSH Terms] OR premenstrual tension[MeSH Terms] OR syndrome, premenstrual[MeSH Terms] OR “prevalence of Premenstrual Syndrome”[Title/Abstract] OR Premenstrual dysphoric disorder[Title/Abstract]) AND (prevalence[Title/Abstract] OR prevalence[MeSH Terms] OR analysis, cross sectional[MeSH Terms] OR “Cross sectional”[Title/Abstract] OR Observational[Title/Abstract] OR “Case control”[Title/Abstract] OR Cohort[Title/Abstract]) AND (Indi*[Title/Abstract] OR “Indian state”[Title/Abstract]). Similar search terms were used in combination for Cochrane library. For Scopus and IndMed, only free text searches with or without truncations were used. We did not impose any language or date restrictions and filters while running the search. Major Indian journals on the concerned topic were also consulted to identify any additional, relevant studies. Finally, the snow balling method of search was adopted through the screening of bibliographic list of relevant papers for any additional, relevant studies.

### Selection of studies

Two reviewers (AD & AS) were independently involved in screening of the retrieved studies for titles and abstracts. After the preliminary screening, full texts of the relevant studies were examined according to the following eligibility criteria:

1. Studies primarily reporting extractable prevalence data of PMS or PMDD
2. Studies conducted in any part of India and not on mixed population.
3. Study types included cross-sectional studies, case-control studies or cohort studies (only baseline data were analysed)
4. Studies published after the year 2000 and reporting the population being studied adequately.

We excluded editorials, letters, commentaries, studies with inadequate data, reviews, posters, and interventional studies, preprint documents and grey literature (including theses).

Any confusion or discrepancy regarding the selection of studies was resolved by consensus between the authors. If a single study was reported in multiple publications, then only the one with comparatively better quality and larger sample size was included for analysis.

### Quality assessment 

All the retrieved studies were evaluated for methodological quality by two reviewers (AD & AS) independently, using a modified version of Newcastle Ottawa Scale (NOS).^16^ We considered five domains (1 point each) for assessing quality of each study: “sample representativeness,” “sample size,” “ascertainment of Premenstrual syndrome,” “comparability between respondents and non-respondents,” and “statistical quality”.

According to the resultant number of points assigned, each study was judged to be at high (≥ 3 points) or low (< 3 points) quality. Any discrepancies concerning the author’s judgments were resolved by consensus.

### Data extraction

One reviewer extracted the data (AD), which was cross-checked by the other (AS). Following data were extracted in a standardised excel sheet for each eligible study: Author, year of publication, study region, time frame of data collection, age range of the study participants, study population, diagnosis, diagnosis criteria or tools used, sample size and reported prevalence. In case of incomplete or incomprehensible data, the concerned author was contacted for clarification and non-response was considered as drop-out. Any disagreements between the authors were resolved by discussion.

### Statistical analysis

Meta-analysis was carried out to estimate the prevalence pooled from individual studies. Summary estimates were reported along with their 95% CIs, for both PMS and PMDD. Heterogeneity between studies was quantified with the I^2^ statistic. In general, I^2^ values of 25%, 50% and 75% are considered as belonging to, low, moderate and high category of heterogeneity respectively.^17^ According to the presence of heterogeneity between prevalence studies, we used fixed or random effect model following DerSimonian and Laird’s method.

Subgroup analyses according to the States/UTs, quality of the studies (high vs. low), and population (adolescent vs. mixed population) were carried out. We also ran sensitivity analysis to evaluate the effect of individual studies on the pooled estimates. All the analyses were done using *‘metafor’ and*‘*metaprop’* package in ‘R’ software (https://cran.r-project.org/). Presence of publication bias was evaluated through visual inspection and rank correlation test for any asymmetry present in the funnel plot.

## Results

### Search results and study characteristics

**Figure 1:**
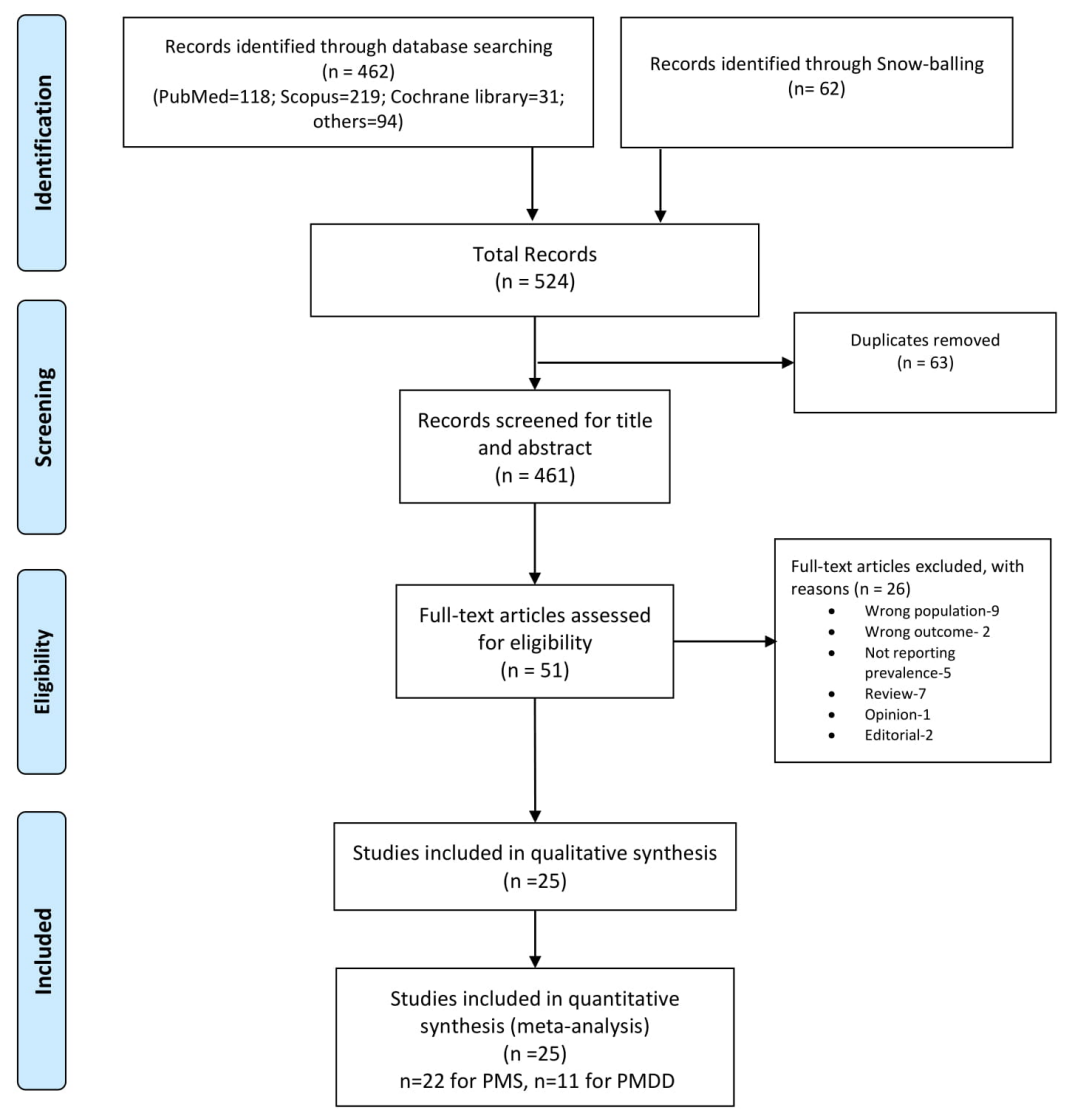
Study selection process.

The final search yielded 524 citations including snowballing; following the removal of 63 duplicate records, 431 abstracts were screened for potential eligibility. Of remaining 51 studies (full-text screening), 26 were excluded on the basis of specific article types and eligibility. Finally 25 studies were included for the systematic review and meta-analysis. The study selection process is depicted in detailed manner in Figure 1.

Among 25 included studies, all were of cross-sectional design and reported from different states at varied number and time points. Very few studies reported community-based prevalence, mostly reporting prevalence from school or college settings. Collective number population was 8542; sample size ranged from 60 to 1281, with a median of n=224. We found only three studies published before 2011, and four studies on adolescence age group (if we strictly consider adolescents up to the age of 19). Three studies exclusively reported PMDD, whereas eight evaluated both PMS and PMDD; 22 were exclusively on the prevalence of PMS. A detailed description of included studies is provided in Table 1.

### Quality of included studies

Owing to all included studies being of cross-sectional design, we used modified version of NOS for evaluation of the quality of each included study. The majority of studies (n=14) are rated as high,^9,11,12,18-28^ while the remaining 11 studies were evaluated as low quality.^10,29-38^ Studies were mostly at low risk of bias regarding assessment of outcome and statistical tests. However, the studies were at high risk of bias in terms of sample representativeness, sample size and response rate/reporting (Table 2).

### Factors related with PMS and PMDD

The influence of the presence of various risk factors on PMS or PMDD were explained or analysed in 13 of 25 studies. The increased prevalence of PMS or PMDD was associated with the consumption of tea, coffee, sweet or sweetened beverages, junk food and food intake under stress.^12,25^ Several studies also reported positive correlation with the lack of physical activity or leading a sedentary lifestyle.^12,25,29,33^ Bhuvaneswari et al^12^ reported that a positive family history of PMS/PMDD was associated with higher prevalence, while Laxmi et al^34^ found no significant association, whereas Badkur et al^31^ reported negative association with the history of menstrual problems in mother. The bitter receptor gene TAS2R38 was found to be correlated with severity and susceptibility of PMS.^21^ Among psychological variables, increased stress^33,38^ was the most important followed by anxiety, depression.^38^ However, socio-demographic variables like age, body mass index (BMI), amount of blood flow during menstruation, dysmenorrhoea, age of menarche and place of residence were reported as having either positive^20,24,27,33,37^ or no correlation.^10^

### Prevalence of PMS

**Figure 2:**
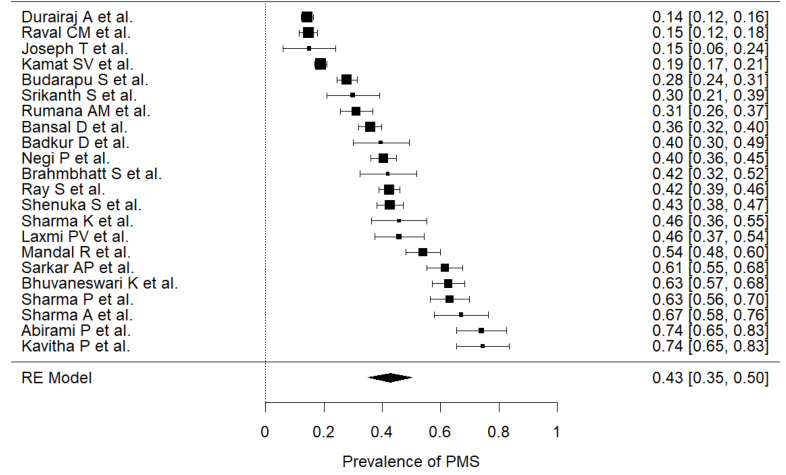
Pooled Prevalence of premenstrual syndrome. Random-Effect model; Heterogeneity statistics: I^2^=98.26%, Q=1205.976, *P* < 0.001.

The prevalence of PMS was reported in 22 studies,^9-12,18-24,26-36^ including n=7865 participants (median=257), across different states. Participants were recruited from various sampling frame using different sampling methods, which has a very low chance of overlapping and overweighing of results. The estimated prevalence (Figure 2), pooled from all included studies was found to be 43% (95% CI: 0.35-0.50) with a high level of heterogeneity (I^2^=98%). The highest prevalence was found by pooling studies reported from Delhi, estimated at 64.4% (95% CI: 0.59-0.70, I^2^=0) and lowest was for Kerala at 15.3% (95% CI: 0.69-0.25).

**Figure 3:**
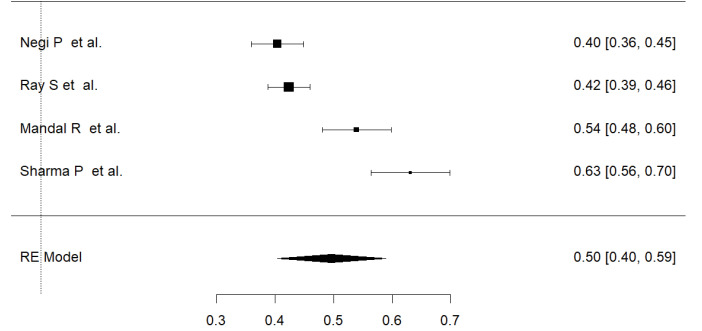
Pooled prevalence of premenstrual syndrome among adolescents. Random-Effect model; Heterogeneity statistics: I^2^=92.84%, Q=41.899, *P* < 0.001.

We also estimated the prevalence of PMS among adolescents (considering the age limit 10-19 years) from data extracted from four studies (Figure 3). The overall estimated prevalence of PMS among Indian adolescents was 49.6% (95% CI: 0.40-0.59, I^2^=93%).

Sensitivity analysis was performed to check the effect of individual study on the overall estimate. No study had significant effect on changing the estimate more than 2%, thus confirming the robustness of the estimated prevalence.

### Prevalence of PMDD

A total of 11 studies^9,11,12,25,30,32,33,35-38^ with 5578 participants (median=478) were included in the meta-analysis. Prevalence reported by individual studies were ranged from 3.7% to 65.67%. The pooled estimate of PMDD was 14% (95% CI: 0.10-0.21) with a heterogeneity, I^2^=98.32%.

**Figure 4:**
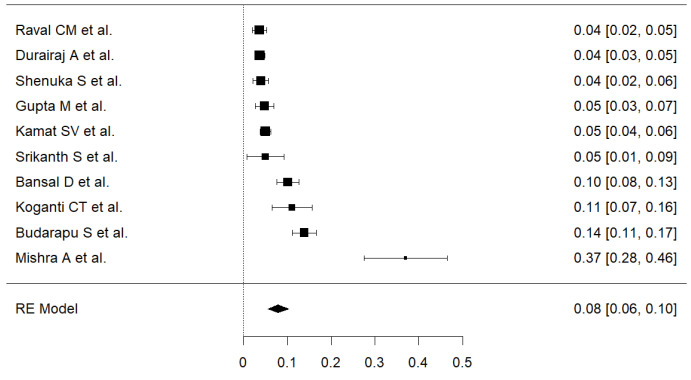
Pooled prevalence of premenstrual dysphoric disorder. Random-Effect model; Heterogeneity statistics: I^2^=92.28%, Q=116.541, *P* < 0.001.

In sensitivity analysis, exclusion of a study by Bhuvneswari et al,^12^ dropped the overall prevalence significantly. The estimated prevalence of rest of the 10 studies was 8% (95% CI: 0.06-0.10), but still the heterogeneity remained high (I^2^=98.32). Further analysis did not show sensitivity of the overall estimate for any study more than 2%. Therefore, the latter estimate was more robust and taken into consideration (Figure 4). A sensitivity analysis using double arcsine transformation showed similar results.

### Subgroup analysis

A further subgroup analysis was carried out in terms of prevalence from individual State/UT, and quality of the included studies (Tables 3 and 4). We found studies from 10 States/UTs of India reporting prevalence of PMS, of these, a very small number of studies were conducted at the community level. Studies from Delhi showed the highest prevalence of PMS [64.4% (95% CI: 58.9-69.7, I^2^=0%)], whereas Kerala showed the lowest [(15.3% (95% CI: 6.9-25.3)]. The estimated prevalence of PMDD was maximum at Puducherry, at 65.7% (95% CI: 0.60-0.71) and minimum for Chandigarh, 4.8% (95% CI: 0.03-0.07). Notably, both the estimates for PMDD were based on the single studies. However, prevalence estimate of PMDD for Tamil Nadu based on two studies, was most robust in our study, at 3.8% (95% CI: 0.03-0.05) with heterogeneity, I^2^=0%.

The difference of pooled estimates between high and low quality studies were different. For PMS, the difference between low and high quality of studies was 4.6%. But, in case of PMDD, prevalence in ‘low quality subgroup’ was 7.7% higher than the ‘high quality’ subgroup. However, even after excluding the ‘low quality studies,’ the overall estimate of PMDD did not change much.

**Figure 5:**
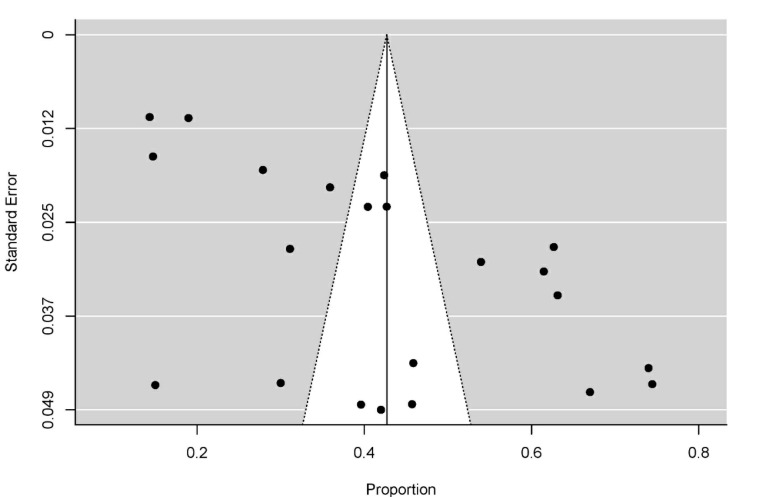
Funnel plot.

Publication bias was not indicated through the visual inspection of the funnel plot (Figure 5) and ‘rank correlation test for Funnel plot asymmetry’ (Kendall’s Tau = 0.186; *P* = 0.239)

## Discussion

This is the first systematic review and meta-analysis that has comprehensively searched and synthesised studies reporting prevalence of PMS and/or PMDD in the Indian population. In this study, we found that both the conditions are common among Indian females. The pooled prevalence estimates of PMS and PMDD among Indian females were 43% (95% CI: 0.35-0.50) and 8% (95% CI: 0.06-0.10), respectively. The prevalence varied with the geographic region, with the highest prevalence of PMS being reported in Delhi, whereas the lowest prevalence was reported in Kerala.

We observed a substantial heterogeneity in meta-analyses of both the estimates, which might be explained by age group, geographical region, study settings, residence in a rural or urban area, type of diagnostic tool and cut-off points used, or the study quality. We surmise from the report of different studies that substantial presence of factors such as socioeconomic conditions, diet, genetic pattern, family history, custom could have some role.

It may be instructive to look at prevalence estimates of premenstrual disorders in other low- and middle-income countries. A meta-analysis reported the pooled prevalence of PMS in Iran^39^ was 70.8% (95% CI: 63.8-77.7) which is much higher than the present findings for India. Similarly, Chandraratne and Gunawardena^40^ found that 65.7% of adolescents from Sri Lanka experienced PMS, where the commonest somatic symptom was fatigue, and a study on Pakistani women^41^ reported a prevalence of 79.9%. Our findings are quite similar to a cross-sectional study conducted in Taiwan which found a prevalence of PMS at 39.85% in female university students.^42^ Other studies from neighbouring countries showed lower prevalence of PMS – prevalence of 37.3% was reported in a study on the reproductive aged women of Myanmar^43^ and 21.1% in a community-based sample in China.^44^

The pooled prevalence of PMDD was also higher in India than a community-based study from China^44^ which reported 2.1%, and in a study from Pakistan^41^ prevalence was 5.5%. However, a recent meta-analysis of studies on PMDD among adolescents of Ethiopia^45^ reported a prevalence of 54.5%, which is much higher than our estimate.

This systematic review and meta-analysis is the first of its kind to estimate the national burden of PMS and PMDD in Indian population. This study is significant in terms of rigorous search in national and international databases along with snowballing through hand-search within different relevant articles minimising chances of missing important relevant studies. A systematised protocol was registered beforehand in the international prospective register of systematic reviews (PROSPERO) to maintain transparency throughout the study process. However, we included PMDD along with PMS as the topic of interest as they lie in the same continuum, which was not or faintly decided during protocol stage, but found important in the course of the study. The standard nature of this review was maintained by adhering to the PRISMA and MOOSE guidelines throughout different steps. An important caveat while interpreting the results is the presence of substantial amount of heterogeneity. However, this type of heterogeneity is expected due to the demographic variation, cultural differences and other factors in a country as diverse as India. The reason for such heterogeneity was attempted to be explained by means of sensitivity analysis and subgroup analysis. We assessed the quality of each study with a modified version of NOS and ran a subgroup analysis to assess any influence of it on the results. Most of the included studies were not conducted in community settings which might have affected reaching a precise estimate. Different institutional studies are expected to have included a mixed population from various states or regions. In sensitivity analysis, we confirmed the robustness of the overall obtained estimate which might have increased the generalizability of the results. The study by Bhuvaneswari et al^12^ influenced the overall estimate of PMDD to a greater degree, probably due to false high estimate, as reported, so we calculated the overall prevalence after excluding the study. Owing to the small number of studies belonging to each region, the state-wise prevalence must be interpreted with caution.

## Conclusion

We found a high prevalence of PMS and PMDD among Indian females of reproductive age group. A comparatively high prevalence was found among adolescents which affects the quality of life adversely. These are largely contributed by various socio-demographic, genetic and psychological factors. Owing to the less number of studies and sharp variation of estimates between regions, large scale studies (preferably nationwide) are required to find out more robust estimates of disease burden and potentially associated factors. However depending on the present findings, we recommend the development of governmental policies and guidelines to address this problem at individual and community level to increase the health status and productivity of this population.

## Funding

This study received no external funding.

## Competing interests

None.

## Ethical approval

This study only used published secondary data and did not involve any participants directly. Therefore, permission from the human ethics committee was not applicable.

## Authors’ contributions

AD, Conceptualization, Project administration, Supervision, Validation, Literature Search, Methodology, Data curation, Appraisal, Formal analysis, Resources, Software, Visualization, Writing – original draft, Writing – review & editing. AS, Clinical input, Project administration, Supervision, Validation, Interpretation, Writing – review & editing.

## Disclaimer

No part of this paper is copied from other source.

**Table 1:** Summary of included studies

**Author**	**Year**	**Place (State/UT)**	**Data collection period**	**Age (y)**	**Population**	**Diagnosis**	**Scale used/criteria**	**Population studied (N)**	**PMS** ** No. (%)**	**PMDD** ** No. (%)**
Sharma et al^18^	2008	Delhi	-	13-19	Adolescents	PMS	Pre-tested, semi-structured questionnaire	198	125 (63.13)	-
Sharma et al^19^	2008	Delhi	-	20-23	Unmarried undergraduate medical students	PMS	12 Symptoms of PMS	100	67 (67)	-
Ray et al^20^	2010	West Bengal	-	10-19	Adolescents	PMS	Pre-tested questionnaire	715	303 (42.38)	-
Sharma et al^21^	2013	Himachal Pradesh	-	20–50	Adult women	PMS	PMS (A,D,C,H,O)	105	48 (45.71)	-
Brahmbhatt et al^22^	2013	Gujrat	-		Medical and nursing students, teaching and non-teaching staffs	PMS	Structured questionnaire developed for PMS	100	42 (42)	-
Mandal et al^23^	2015	West Bengal	Jul-Aug, 2014	13-19	Adolescents	PMS	ACOG criteria	278	150 (53.96)	-
Sarkar et al^24^	2015	West Bengal	Jul-Aug, 2015	13–21	Adolescents	PMS	ACOG criteria	244	150 (61.48)	-
Mishra et al^25^	2015	Gujrat	-	19–28	UG and PG Medical students	PMDD	SPAF; SSQ-PMDD	100	-	37 (37)
Kavitha et al^10^	2015	Tamil Nadu	Feb- Apr, 2015	-	Medical students	PMS	DSM-IV	90	67 (74.44)	-
Joseph et al^26^	2016	Kerala	-	18-20	Nursing students	PMS	Modified standardized premenstrual syndrome scale	60	9 (15)	-
Raval et al^11^	2016	Gujrat	Jan- Aug, 2012	17–30	College students	PMS and PMDD	PSST; SCID-PMDD	489	72 (14.72)	18 (3.68)
Rumana et al^27^	2017	Karnataka	-	up to 25	Medical students	PMS	PEQ	270	84 (31.11)	-
Abirami and Ambika^28^	2017	Tamil Nadu	Jan 5-21, 2015	17-26	Nursing students	PMS	Structured questionnaire developed for PMS	100	74 (74)	-
Negi et al^29^	2018	Himachal Pradesh	-	13-19	Adolescents	PMS		470	190 (40.43)	-
Shenuka et al^30^	2018	Tamil Nadu	May- Oct, 2017	17-27	Healthcare and non-healthcare students	PMS and PMDD	PMS (A,D,C,H,O) and PSST	478	204 (42.68)	19 (3.97)
Badkur et al^31^	2018	Madhya Pradesh	Dec- Jun, 2015	18-25	College students	PMS	ACOG criteria	101	40 (39.6)	-
Budarapu et al^32^	2018	Andhra Pradesh	-	-	Medical students	PMS and PMDD	PSST	635	177 (27.87)	88 (13.86)
Kamat et al^33^	2019	Gujrat	-	10–23	College and school students	PMS AND PMDD	PSST-A	1281	243 (18.97)	64 (5)
Bhuvaneswari et al^12^	2019	Puducherry	-	18-22	College students	PMS and PMDD	SPAF for PMS	300	188 (62.67)	197 (65.67)
Laxmi et al^34^	2019	Andhra Pradesh	-	17-24	Nursing students	PMS	Structured questionnaire containing 47 questions of PMS	133	61 (45.86)	-
Durairaj et al^9^	2019	Tamil Nadu	-	17-25	Medical and engineering students	PMS and PMDD	PSST	1047	150 (14.33)	39 (3.72)
Srikanth and Nandini^35^	2019	Andhra Pradesh	-		Medical students	PMS and PMDD	PSST	100	30 (30)	5 (5)
Bansal et al^36^	2019	Karnataka	Sept 2017- Aug 2018	16-30	College students	PMS and PMDD	PSST	571	205 (35.9)	58 (10.16)
Koganti et al^37^	2020	Telengana	Sept- Dec 2019	18-25	Medical students	PMDD	DSR	180	-	20 (11.11)
Gupta et al^38^	2020	Chandigarh	-	11–20	School Students	PMDD	PMDD scale	397	-	19 (4.79)

UT: Union Territory; ACOG: American College of Obstetricians and Gynaecologists; DSM-IV-TR: Diagnostic and Statistical Manual of Mental Disorders, 4th Edition, Text Revision; DSR: Penn State Daily Symptom Report; PEQ: PMS self-Evaluation Questionnaire; PMS (A, D, C, H, O): Premenstrual Syndrome Symptoms (Anxiety, Cravings, Heaviness, Hydration, Headaches, Depression, Others); PSST: Premenstrual Symptoms Screening Tool; PSST-A: Premenstrual Symptoms Screening Tool-Adolescents; SCID-PMDD: Structured Clinical Interview for DSM-IV-TR Defined PMDD; SPAF: Shortened Premenstrual Assessment Form; SSQ-PMDD: Self-screening quiz for PMDD as per the DSM-IV-TR criteria.

**Table 2:** Quality assessment of studies using modified Newcastle Ottawa Scale

**Author**	**Selection**			**Outcome**		**Total score**	**Quality**
	**Sample representativeness**	**Sample size**	**Response rate**	**Assessment of outcome**	**Statistical tests**		
Sharma et al^18^	*	-	*	-	*	3	High
Sharma et al^19^	*	*	-	-	*	3	High
Ray et al^20^	*	*	*	-	*	4	High
Sharma et al^21^	-	-	-	*	*	2	Low
Brahmbhatt et al^22^	-	-	-	-	*	1	Low
Mandal et al^23^	-	-	-	*	*	2	Low
Sarkar et al^24^	*	-	-	*	*	3	High
Mishra et al^25^	-	-	-	*	*	2	Low
Kavitha et al^10^	-	-	-	-	*	1	Low
Joseph et al^26^	*	-	-	-	*	2	Low
Raval et al^11^	-	*	*	*	*	4	High
Rumana et al^27^	-	-	-	*	*	2	Low
Abirami and Ambika^28^	-	-	-	-	*	1	Low
Negi et al^29^	-	*	-	-	*	2	Low
Shenuka et al^30^	*	*	*	*	*	5	High
Badkur et al^31^	*	-	-	*	*	3	High
Budarapu et al^32^	-	*	*	*	*	4	High
Kamat et al^33^	*	*	*	*	-	4	High
Bhuvaneswari et al^12^	-	*	-	*	*	3	High
Laxmi et al^34^	-	-	-	*	*	2	Low
Durairaj et al^9^	-	*	*	*	*	3	High
Srikanth and Nandini^35^	-	-	-	*	-	1	Low
Bansal et al^36^	-	*	*	*	*	4	High
Koganti et al^37^	-	-	*	*	*	3	High
Gupta et al^38^	*	*	-	*	*	4	High

**Table 3:** Subgroup analysis of PMS studies

**Category**	**Groups**	**Prevalence**
Study Quality	High	40.6% (95% CI: 30.9-50.3, I^2^=99%)
	Low	45.2% (95% CI: 35.0-55.4, I^2^=95%)
State or UT	Andhra Pradesh	34.1% (95% CI: 23.3-45.5, I^2^=87%)
	Delhi	64.4% (95% CI: 58.9-69.7, I^2^=0%)
	Gujrat	23.2% (95% CI: 14.2-33.2, I^2^=94%)
	Himachal Pradesh	41.4% (95% CI: 37.4-45.5, I^2^=0%)
	Karnataka	33.9% (95% CI: 29.4-38.6, I^2^=46%)
	Kerala	15.3% (95% CI: 6.9-25.3)
	Madhya Pradesh	39.6% (95% CI: 30.3-49.4)
	Puducherry	62.7% (95% CI: 57.1-68.1)
	Tamil Nadu	50.4% (95% CI: 20.1-80.7, I^2^=99%)
	West Bengal	52.4% (95% CI: 40.6-64.2, I^2^=93%)

**Table 4:** Subgroup analysis of PMDD studies

**Category**	**Groups**	**Prevalence**
Study Quality	High	5.2% (95% CI: 0.04-0.09, I^2^=92%)
	Low	18.4% (95% CI: -0.00-0.58, I^2^=97%)
State or UT	Andhra Pradesh	9.5% (95% CI: 0.02-0.19, I^2^=87%)
	Gujrat	11.6% (95% CI: 0.02-0.24, I^2^=97%)
	Karnataka	10.2% (95% CI: 0.08-0.13, I^2^=96%)
	Telengana	11.1% (95% CI: 0.07-0.16)
	Puducherry	65.7% (95% CI: 0.60-0.71)
	Tamil Nadu	3.7% (95% CI: 0.03-0.05, I^2^=0%)
